# Genome-wide association screening and verification of potential genes associated with root architectural traits in maize (*Zea mays L.*) at multiple seedling stages

**DOI:** 10.1186/s12864-021-07874-x

**Published:** 2021-07-20

**Authors:** Abdourazak Alio Moussa, Ajmal Mandozai, Yukun Jin, Jing Qu, Qi Zhang, He Zhao, Gulaqa Anwari, Mohamed Abdelsamiaa Sayed Khalifa, Abraham Lamboro, Muhammad Noman, Yacoubou Bakasso, Mo Zhang, Shuyan Guan, Piwu Wang

**Affiliations:** 1 College of Agronomy, Plant Biotechnology Center, Jilin Agricultural University, 130118 Changchun, Jilin China; 2 College of Life Sciences, Jilin Agricultural University, Jilin 130118 Changchun, China; 3 Biology Department, Faculty of Sciences and Techniques, Abdou Moumouni University of Niamey, 10662 Niamey, Niger

**Keywords:** Maize, root-related traits, GWAS, SNPs, Candidate genes, qRT-PCR

## Abstract

**Background:**

Breeding for new maize varieties with propitious root systems has tremendous potential in improving water and nutrients use efficiency and plant adaptation under suboptimal conditions. To date, most of the previously detected root-related trait genes in maize were new without functional verification. In this study, seven seedling root architectural traits were examined at three developmental stages in a recombinant inbred line population (RIL) of 179 RILs and a genome-wide association study (GWAS) panel of 80 elite inbred maize lines through quantitative trait loci (QTL) mapping and genome-wide association study.

**Results:**

Using inclusive composite interval mapping, 8 QTLs accounting for 6.44–8.83 % of the phenotypic variation in root traits, were detected on chromosomes 1 (qRDW_v3_-1-1 and qRDW/SDW_v3_-1-1), 2 (qRBN_v1_-2-1), 4 (qSUA_v1_-4-1, qSUA_v2_-4-1, and qROV_v2_-4-1), and 10 (qTRL_v1_-10-1, qRBN_v1_-10-1). GWAS analysis involved three models (EMMAX, FarmCPU, and MLM) for a set of 1,490,007 high-quality single nucleotide polymorphisms (SNPs) obtained via whole genome next-generation sequencing (NGS). Overall, 53 significant SNPs with a phenotypic contribution rate ranging from 5.10 to 30.2 % and spread all over the ten maize chromosomes exhibited associations with the seven root traits. 17 SNPs were repeatedly detected from at least two growth stages, with several SNPs associated with multiple traits stably identified at all evaluated stages. Within the average linkage disequilibrium (LD) distance of 5.2 kb for the significant SNPs, 46 candidate genes harboring substantial SNPs were identified. Five potential genes viz. Zm00001d038676, Zm00001d015379, Zm00001d018496, Zm00001d050783, and Zm00001d017751 were verified for expression levels using maize accessions with extreme root branching differences from the GWAS panel and the RIL population. The results showed significantly (*P* < 0.001) different expression levels between the outer materials in both panels and at all considered growth stages.

**Conclusions:**

This study provides a key reference for uncovering the complex genetic mechanism of root development and genetic enhancement of maize root system architecture, thus supporting the breeding of high-yielding maize varieties with propitious root systems.

**Supplementary Information:**

The online version contains supplementary material available at 10.1186/s12864-021-07874-x.

## Background

Maize *(Zea mays L.)* is one of the most widely produced grain crops in the world [1]. With the fast-growing world population, improving the yield of corn has become an important target for breeders. The root system plays a primordial role in plant species growth and development and even productivity [2–4]. Plants rely on the root system for anchorage and the acquisition and absorption of nutrients essential for sustaining productivity [2]. As the place of plant and soil interactions, roots play a fundamental role in plant responses to biotic and abiotic stresses [5], and influence significantly many agronomically important traits, including drought and flood tolerance [6–8], root-lodging resistance [9], and nutrient use efficiency particularly nitrogen (N), phosphorus(P), and calcium (Ca) under suboptimal growth conditions [10–13] and resource-challenging environments [2, 14, 15]. Important synchronizations were previously revealed between root growth especially shoot-borne roots with N uptake efficiency in maize [15, 16]. Besides, some pieces of evidence support that high yielding maize varieties are supposed to have propitious root systems, which may efficiently sustain water and nutrients, resulting in increased yield [17] especially under limited water or nutrient availability [18]. Furthermore, grain yield was reported to be closely correlated with root related traits in the early stages of maize development [19]. Nevertheless, maize roots have received much less attention than shoot structures since they are hidden, complex, dynamic and greatly influenced by the soil environment [15, 20–22]. Due to the challenge in achieving reliable root-related trait data from the field, characterizing crops such as maize with improved root system characteristics in the field remains still a major challenge to current plant biology [18, 23] and root trait phenotyping studies commonly use soil-less nutritive solutions [5]. However, it was previously indicated that plant growing systems that nearly mimic the soil media are more stable in mineral elements and environmental factors, and easier to operate for root morphological traits phenotyping in maize [24]. Therefore, to offer a better and robust tool for plant behavior prediction under field conditions, various experimental growing systems with soil-based substrates have been implemented [25–28]. Owing to the rapid progress in digitally automatic image analysis, root phenotypic data acquisition is becoming nowadays cheaper, quicker and more effective [29–34]. Thus, numerous software frameworks such as ARIA [31], EZ-Rhizo [35], Smart Root [36], WinRhizo [37], Optimas analysis software, Image J [38], Root Nav [32], IJ_Rhizo [39], Root System Analyzer [40], and Root Trace [41] have been broadly used for automated root traits measurements in a high throughput manner.

To date, several Quantitative Trait Loci (QTL) studies have been conducted to locate root-trait QTLs under various conditions of growth, at diverse developmental stages and involving various genetic populations [21, 22, 24]. Yet, due to low-density markers and large confidence intervals, the localizations of the identified QTLs were inconsistent among the different findings. Thus, further root studies were necessary to detect more chromosomal regions and ultimately identify consistent loci to further screen and verify candidate genes crucial for marker-assisted selection. By enabling the identification of essential loci at high-resolution, association studies have several advantages over conventional genetic mapping approaches for understanding the genetic basis of complex traits [42] like root traits in maize [2]. In the 21st century, genome-wide association studies (GWAS) have been auspiciously used as a high-throughput technique to analyzing the genetic basis for a variety of major crops [42], such as rice, sorghum, soybean, wheat, and maize essential for modern genetic studies [43]. Recently, Sanchez et al. [44] used 300 doubled haploid exotic introgression lines and found 39 SNPs for root architecture traits along with, multiple SNPs within candidate genes that displayed expression in maize seedling roots. In a GWAS analysis implying 14 days old maize seedlings generated from 384 inbred lines genotyped by sequencing (GBS), 268 SNPs associated with root morphological traits, along with 9 SNPs within one candidate gene region were reported [2]. Zaidi et al. [8] used a CIMMYT Asia panel involving 396 tropical maize lines. They revealed 67 SNPs associated with root structural traits under drought stress with many SNPs found within various candidate gene regions. However, to our knowledge no previous study has investigated the genetic basis of maize root architectural traits at multiple developmental stages with successful functional verification of associated candidate genes. Therefore, the objectives of this study were to (i) screen the existing phenotypic variability of root architectural traits within a maize elite germplasm at multiple seedling stages, (2) detect novel significant genomic regions throughout the whole genome and across stages associated with root architectural traits, and (3) identify and verify the expression of possible potential candidate genes.

## Results

### Phenotypic analysis of root architectural traits

From the mapping population, the evaluated root traits displayed large variations both in parental lines and their offspring at all the three stages (Table 1 and Additional file 1: Table S1). Analysis of variance related to the root trait performances of the two parental lines revealed significant to highly significant differences (*P* < 0.05; *P* < 0.01; *P* < 0.001) for all the measured seedling traits and at all the three stages except RDW/SDW at V1 stage (Table 1). Comparing the two parents, P014 displayed significantly higher root trait performances than E1312 across the three stages (Table 1). This result shows the instantaneous nature of the development of the two parental root systems over time which confirms the pertinence of the three selected experimental time-points for root traits assessment. RBN and TRL exhibited the largest variations of 264.94 and 121.00 cm, respectively (Table 1). Similar heritability and correlation patterns were also observed across stages. Heritability values ranged between 50.22 ( for RDW/SDW) and 99.96 % (for TRL). SUA and TRL exhibited the strongest positive significant correlations (*r* = 0.924; *P* < 0.01) while RDW/SDW showed very weak correlations with all other traits (*r* = 0.149 ~ 0.464; *P* < 0.05; *P* < 0.01; Table 2).

**Table 1:** Descriptive statistics of the seven root-related traits within the mapping population at V1, V2 and V3 stages

Traits	Stage	P014	E1312	Sig.^a^	RILs						
		Mean	Mean		Mean	±SD	Range	Skewness	Kurtosis	CV (%)	H^**2**^(%)
RDW(g)	V1	0.04	0.01	*	0.03	0.03	0.17	1.85	3.60	90.05	70.91
	V2	0.06	0.03	**	0.05	0.03	0.20	1.37	2.58	59.01	69.34
	V3	0.07	0.04	**	0.10	0.05	0.32	1.06	1.32	52.54	85.73
RDW/SDW	V1	0.87	0.38	ns	0.78	1.10	9.91	4.28	22.43	141.33	65.94
	V2	0.48	0.38	**	0.33	0.19	2.28	3.66	26.67	58.90	50.22
	V3	0.25	0.19	*	0.41	0.17	1.49	1.25	3.56	41.91	83.27
TRL(cm)	V1	73.79	36.86	***	55.31	25.19	147.89	0.68	0.68	45.55	98.81
	V2	155.29	100.07	***	123.57	57.78	418.32	1.46	4.41	46.76	99.24
	V3	216.63	107.19	***	249.96	121.00	521.61	0.44	-0.69	48.41	99.96
SUA (cm^2^)	V1	28.19	16.42	***	19.32	9.75	66.03	1.17	2.93	50.48	99.31
	V2	40.69	35.71	***	40.17	22.12	134.38	1.44	2.88	55.07	94.73
	V3	76.30	43.73	**	91.16	51.44	235.91	0.68	-0.28	56.43	97.89
ARD (mm)	V1	1.13	1.00	**	0.94	0.20	1.46	1.22	4.07	21.46	97.74
	V2	2.31	1.16	***	1.12	0.25	2.32	2.43	11.89	22.52	88.49
	V3	3.49	2.45	***	1.38	0.45	4.57	4.22	28.76	32.69	95.46
ROV (cm^3^)	V1	1.15	0.63	***	0.56	0.36	2.60	2.31	8.84	65.52	98.15
	V2	2.88	0.84	***	1.13	0.73	4.38	1.29	1.91	64.32	96.66
	V3	3.08	1.79	***	2.82	2.26	22.94	3.63	25.17	80.25	97.35
RBN	V1	51.33	28.33	***	45.66	26.57	126.00	0.67	0.03	58.19	99.28
	V2	94.33	52.67	***	105.88	93.84	1051.00	6.33	59.19	88.64	99.79
	V3	331.00	145.33	***	264.94	362.10	4518.00	9.39	107.55	136.67	99.89

*RDW* root dry weight, *RDW/SDW* root per shoot dry weight, *TRL* total root length, *SUA* surface area, *ARD* average root diameter, *ROV* root volume, *RBN* root branching number, *SD* Std. dev, *CV* coefficient of variation, *H*^*2*^ Broad-sense heritability

^a^level of significance via student-test with, *ns* no significant difference

*significantly different at *P* < 0.05

**significantly different at *P* < 0.01

***significantly different at *P* < 0.001

**Table 2:** Pearson correlation coefficients between the seven root-related traits within the mapping population at V1, V2 and V3 stages

Traits	RDW	RDW/SDW	TRL	SUA	ARD	ROV
V1						
RDW/SDW	0.719^**^					
TRL	0.330^**^	0.022				
SUA	0.389^**^	0.081	0.886^**^			
ARD	0.194^**^	0.050	0.055	0.261^**^		
ROV	0.425^**^	0.103	0.669^**^	0.854^**^	0.514^**^	
RBN	0.299^**^	0.050	0.835^**^	0.781^**^	0.087	0.597^**^
V2						
RDW/SDW	0.648^**^					
TRL	0.659^**^	0.270^**^				
SUA	0.535^**^	0.184^**^	0.826^**^			
ARD	0.004	0.029	0.011	0.309^**^		
ROV	0.459^**^	0.156^*^	0.614^**^	0.884^**^	0.502^**^	
RBN	0.473^**^	0.304^**^	0.783^**^	0.758^**^	0.114	0.577^**^
V3						
RDW/SDW	0.675^**^					
TRL	0.771^**^	0.412^**^				
SUA	0.818^**^	0.464^**^	0.924^**^			
ARD	0.000	0.054	-0.036	0.051		
ROV	0.639^**^	0.353^**^	0.680^**^	0.843^**^	0.247^**^	
RBN	0.310^**^	0.149^*^	0.442^**^	0.523^**^	0.169^*^	0.800^**^

*RDW *root dry weight, *RDW/SDW* root per shoot dry weight, *TRL *total root length, *SUA *surface area, *ARD *average root diameter, *ROV *root volume, *RBN *root branching number

the symbol * and ** indicate respectively, significance at *P* < 0.05 and at P < 0.01

**Fig. 1:**
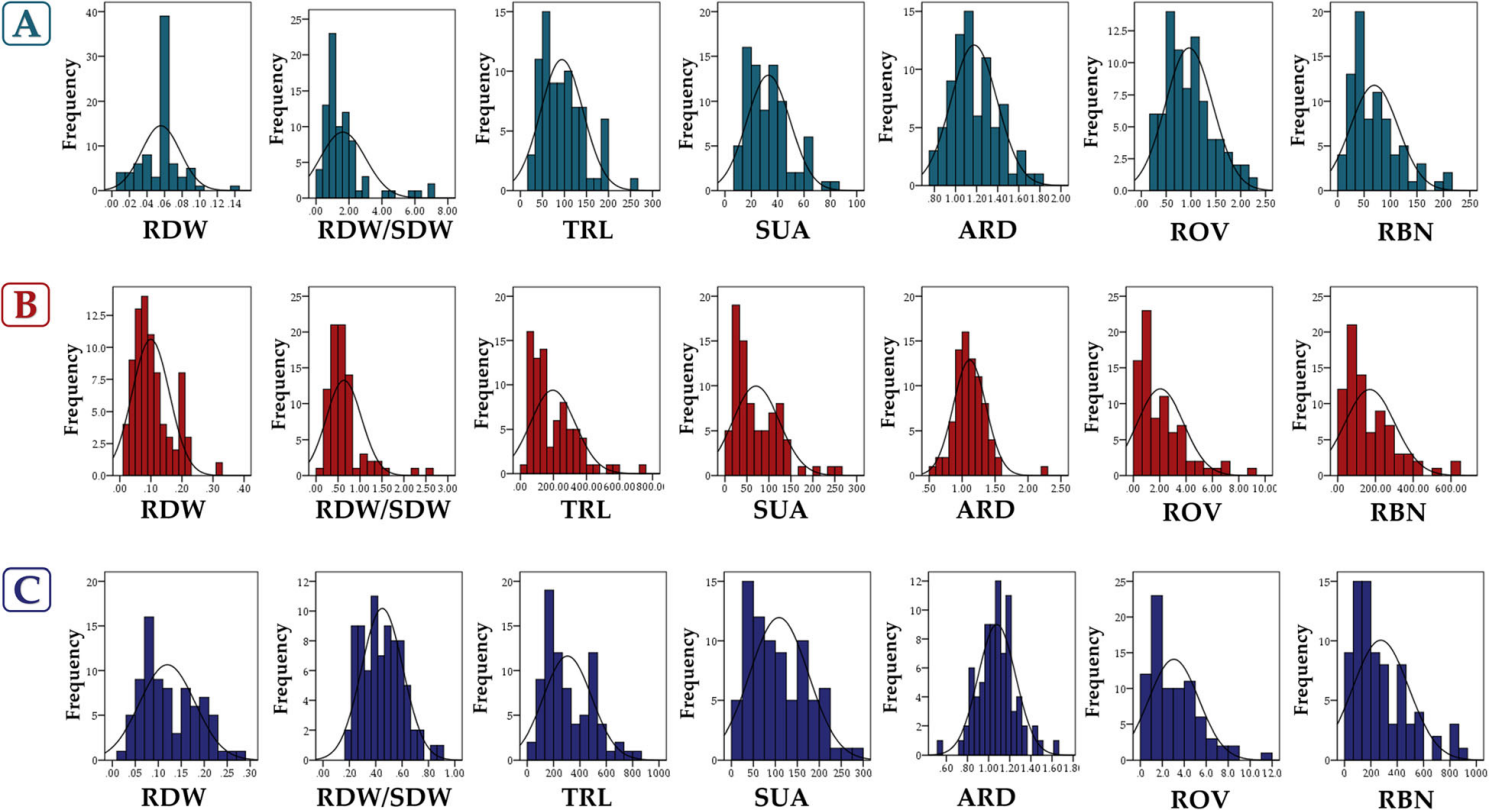
Distribution frequencies of the seven seedling root traits in the GWAS population across three stages (**A, B,** and **C** represent the results from V1 (in turquoise color), V2 (in red color), and V3 (in blue color) stages, respectively). RDW = root dry weight; RDW/SDW = root per shoot dry weight; TRL = total root length; SUA = surface area; ARD = average root diameter; ROV = root volume; RBN = root branching number

Similarly, substantial variation at all growth stages was observed within the GWAS population for all root traits evaluated (Table 3 and Additional file 2: Table S2). At stage V3, RBN and ROV showed the highest coefficients of variation of 77.16 and 76.47 %, respectively. Most root-related traits investigated nearly followed a normal distribution, somewhat skewed from left to right (Fig. 1). Moderate to high broad-sense heritability estimates were observed for all seedling traits and at all stages (Table 3). The highest value was observed from RBN (99.84 %) while the lowest one from ARD (43.20 %) (Table 3). Pearson correlation analysis was also performed to examine the phenotypic relationships among root related traits at each specified stage. Similar significant correlation patterns, but with a greater extent at later stages were detected (Table 4). In regards to all evaluated traits at all stages, ROV and SUA exhibited the strongest positive significant correlations (*r* > 90 %, *P* < 0.01) while RDW/SDW is weakly correlated to all other traits (*r* = -0.199 ~ 0.477; *P* < 0.05; *P* < 0.01; Table 4).

**Table 3:** Descriptive statistics of seedling root related traits for the GWAS population at three stages

Traits	Stage	Mean	±SD	Range	Skewness	Kurtosis	CV (%)	H^2^ (%)
RDW (g)	V1	0.06	0.02	0.15	0.39	2.24	40.82	94.07
	V2	0.10	0.06	0.31	0.97	0.49	62.46	84.68
	V3	0.12	0.07	0.34	0.82	0.33	55.73	71.89
RDW/SDW	V1	0.60	1.38	7.88	2.56	7.39	86.32	90.56
	V2	0.63	0.41	3.27	2.75	11.75	65.32	80.87
	V3	0.45	0.18	1.09	0.80	1.13	39.84	61.71
TRL(cm)	V1	94.29	48.98	261.42	0.93	0.64	51.95	97.87
	V2	195.30	134.37	699.97	1.41	2.45	68.80	99.52
	V3	305.10	184.01	780.26	0.71	-0.33	60.31	99.51
SUA (cm^2^)	V1	32.90	16.67	79.18	0.95	0.54	50.68	96.17
	V2	70.28	52.53	251.41	1.34	1.89	74.74	98.67
	V3	107.91	66.56	294.09	0.66	-0.42	61.68	99.30
ARD (mm)	V1	1.08	0.26	1.46	0.61	0.20	22.11	58.08
	V2	1.12	0.30	2.35	1.88	7.56	27.11	51.31
	V3	1.28	0.22	1.42	0.64	1.29	20.26	43.29
ROV (cm^3^)	V1	0.96	0.52	2.57	0.93	0.63	54.24	75.11
	V2	2.04	1.75	8.83	1.44	1.99	86.06	94.27
	V3	3.02	2.31	13.48	1.35	2.79	76.47	89.26
RBN	V1	69.47	45.45	201.00	1.23	1.15	65.43	98.51
	V2	169.39	132.69	642.00	1.47	2.30	78.34	99.28
	V3	275.04	212.22	864.00	1.18	0.75	77.16	99.84

*RDW* root dry weight, *RDW/SDW* root per shoot dry weight, *TRL* total root length, *SUA* surface area, *ARD* average root diameter, *ROV* root volume, *RBN* root branching number, *SD* Std. dev, *CV* coefficient of variation, *H*^*2*^ Broad-sense heritability

**Table 4:** Pearson correlations at three stages among all root-related traits for GWAS population

Traits	RDW	RDW/SDW	TRL	SUA	ARD	ROV
**V1**						
RDW/SDW	0.509**					
TRL	0.115	-0.251*				
SUA	0.183	-0.224*	0.902**			
ARD	0.037	0.025	-0.303**	-0.031		
ROV	0.172	-0.203	0.731**	0.907**	0.0262*	
RBN	0.104	-0.212	0.752**	0.790**	-0.114	0.749**
**V2**						
RDW/SDW	0.101					
TRL	0.788**	-0.183				
SA	0.822**	-0.214*	0.941**			
ARD	0.381**	-0.127	0.247*	0.418**		
ROV	0.816**	-0.199*	0.857**	0.970**	0.542**	
RBN	0.634**	-0.209*	0.837**	0.852**	0.378**	0.814**
**V3**						
RDW/SDW	0.615**					
TRL	0.773**	0.423**				
SUA	0.843**	0.477**	0.870**			
ARD	0.394**	0.215*	0.104	0.343**		
ROV	0.813**	0.438**	0.704**	0.933**	0.532**	
RBN	0.719**	0.451**	0.900**	0.806**	0.100	0.649**

*RDW *root dry weight, *RDW/SDW *root per shoot dry weight, *TRL *total root length, *SUA *surface area, *ARD *average diameter, *ROV *root volume, *RBN *root branching number

the symbol * and ** indicate respectively, significance at *P* < 0.05 and at *P* < 0.01

### QTL mapping

The linkage map contained 4235 high-quality SNP markers covering a total length of 1514.57 cM distributed for 10 linkage groups [45]. Using inclusive composite interval mapping method with LOD ≥ 2.5 as a threshold, a total of eight substantial QTLs with a phenotypic variance explained ranging from 6.44 to 8.83 % were detected across the three stages (Table 5). The mapped QTLs were allocated to chromosomes 1, 2, 4, and 10. Chromosome 4 contained the highest number of QTLs, with 3 QTLs detected while chromosomes 1, 2, and 10 contained between 1 and 2 QTLs (Table 5). Four QTLs were detected at V1 while two QTLs where identified at both V2 and V3 stages. When examining the number of QTL inheriting parental favorable alleles, the alleles involved in increasing root characteristics at four loci belonged to the parent P014. Meanwhile, the paternal line E1312 contributed to the other four loci, underlying, therefore, the imperative implication of the two parents in root features discrimination. QTL clusters were identified on chromosome 1 and 10 at V3 and V1, respectively. The cluster on chromosome 1 (qRDW_v3_-1-1 and qRDW/SDW_v3_-1-1) located within the marker interval Snp3292_Snp3298 was associated with RDW and RDW/SDW at the genetic region 92.5- 95.5 cM. The Cluster on chromosome 10 (qRBN_v1_-10-1 and qTRL_v1_-10-1) detected within the marker interval Snp62466_Snp62578 was significantly associated to RBN and TRL and spanned 50.5–51.5 cM genetic region. This region harbored two candidate genes GRMZM2G116542 and GRMZM2G016477 predicted to encode a putative Spc97 / Spc98 family of spindle pole body (SBP) component and a putative leucine-rich repeat receptor-like protein kinase, respectively. The three QTLs detected on chromosome 4 (qSUA_v1_-4-1, qSUA_v2_-4-1, and qROV_v2_-4-1) were significantly associated with SUA and ROV and spanned the genetic region 89.5–102.5 cM. QTL qROV_v2_-4-1 (LOD = 3.43, PVE = 8.83 %) associated with ROV was the most significant QTL detected in this study (Table 5). Interestingly, the gene model GRMZM2G068506 predicted to encode a Glucose-1-phosphate adenylyltransferase was found within this chromosomal region. The physical positions of all the detected QTLs are presented in Additional file 3: Table S3.

**Table 5:** Summary of root traits QTLs detected in P014 × E1312 population

QTL^a^	Chr	Bin	Peak(cM)	Marker interval	Genetic interval(cM)	LOD	PVE^b^ (%)	Add.^c^
qRDW_v3_-1-1	1	1.05	95	Snp3292_Snp3298	92.5–95.5	2.51	6.74	-0.01
qRDW/SDW_v3_-1-1	1	1.05	95	Snp3292_Snp3298	92.5–95.5	2.51	6.74	-0.01
qRBN_v1_-2-1	2	2.10	15	Snp16808_Snp16675	14.5–15.5	2.51	6.44	6.38
qSUA_v1_-4-1	4	4.05	91	Snp25452_Snp25434	89.5–91.5	2.67	6.72	2.53
qSUA_v2_-4-1	4	4.05	102	Snp26234_Snp26219	100.5- 102.5	3.03	7.75	5.97
qROV_v2_-4-1	4	4.05	96	Snp25161_Snp25085	95.5–96.5	3.43	8.83	0.21
qTRL_v1_-10-1	10	10.05-06	51	Snp62466_Snp62578	50.5–51.5	2.66	6.77	-6.65
qRBN_v1_-10-1	10	10.05-06	51	Snp62466_Snp62578	50.5–54.5	2.73	7.16	-6.81

^a^The identified QTLs: the name contains trait initials, seedling growing stage, and the number of correspondent chromosome

^b^The percentages of phenotypic variation explained

^c^The QTL additive effect: positive values indicate that P014 provides increased alleles and negative ones indicate that E1312 alleles increased the trait

### NGS analysis results

High-quality genomic data consisted of 3230.75 Gb with an average of 40.38 Gb per sample were obtained from the whole-genome sequencing of the 80 inbred maize lines. All related sequences were made available under the accession number PRJNA495031 in the Sequence Read Archive (https:/www.ncbi.nlm.gov/sra). The averages sequencing depth and coverage were 17.62 and 88.39 %, respectively. With reference to the B73 genome (RefGen_v3), the average similarity rate was 98.82 %.

### Population structure and linkage disequilibrium

**Fig. 2:**
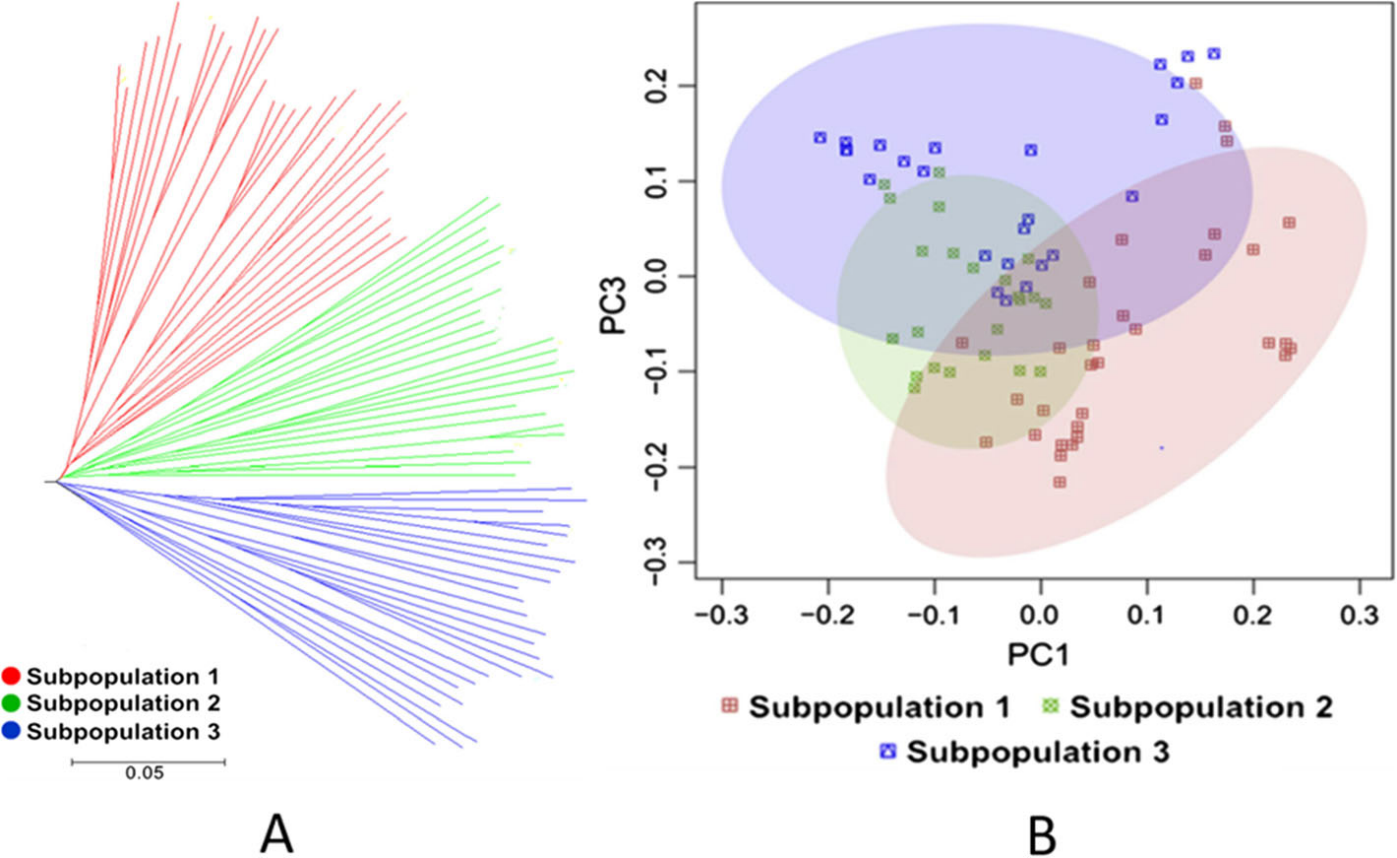
Population structure of the 80 maize accessions: **A** Phylogenetic generated using TreeBeST, **B** Principal component analysis based on genome-wide complex trait analysis software tool (GCTA)

**Fig. 3:**
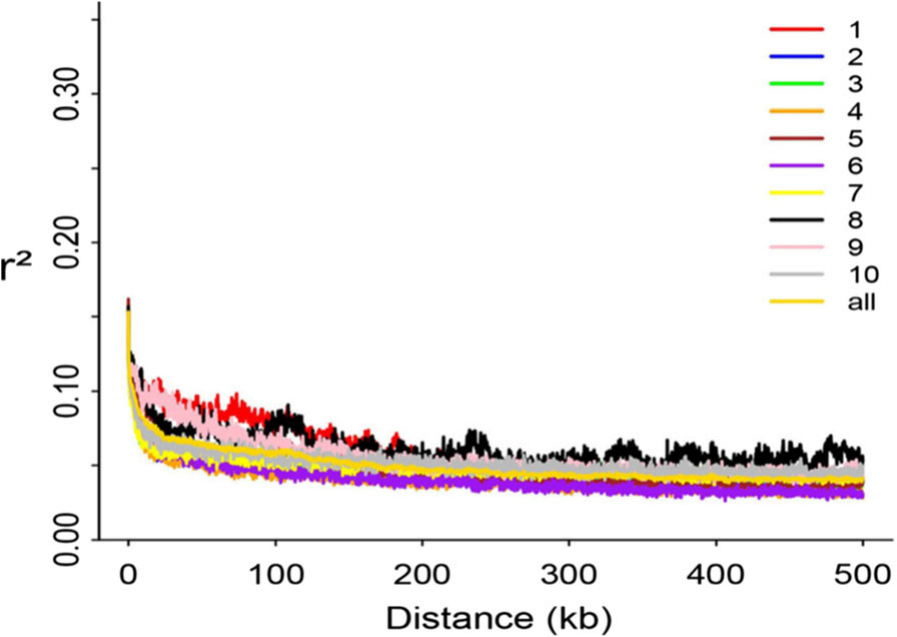
Linkage disequilibrium decay across all 10 maize chromosomes within the 80 maize panel

Based on phylogenetic and PCA analysis, the 80 inbred maize lines were subdivided into three subgroups (Fig. 2A, B). Subgroup 1 mainly included the Reid germplasm represented by PH09B and PH6WC maize inbred lines. Subgroup 2 included mainly the Chinese Lvda Red Cob and Tang Si Ping Tou germplasm as well as some tropical maize lines. Subgroup 3 comprised European and Lancaster germplasm, including Non-Reid maize inbred lines such as PHB1 M and Mo17. As shown in Fig. 3, the average decay distance of the LD across all chromosomes was about 5.2 kb at r^2^ = 0.1. Chromosome 1 with a distance of 10.7 kb showed the highest LD decay while the shortest decay distance (3.7 kb) was observed on chromosome 2.

### GWAS for root architectural traits

**Fig. 4:**
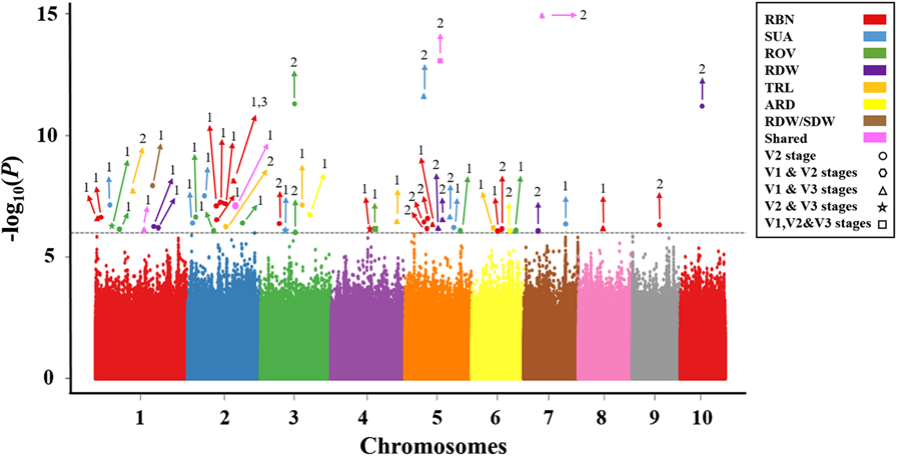
Manhattan plot of all potential significant SNPs associated with each root-related trait at each specific stage (V1, V2, and V3). 1, 2, 3 refer to EMMAX, FarmCPU, and MLM, respectively. RDW = root dry weight; RDW/SDW = root per shoot dry weight; TRL = total root length; SUA = surface area; ARD = average root diameter; ROV = root volume; RBN = root branching number

In this current analysis, three GWAS approaches including EMMAX, FarmCPU, and MLM were used to scan significant SNPs associated with seven root traits namely RDW, RDW/SDW, TRL, SUA, ARD, ROV, and RBN across three vegetative stages (V1, V2, and V3). The detailed list of all significant SNPs detected in this study and their associated genes is presented in Additional file 4: Table S4. SNPs identified within candidate genes or across at least two different stages/methods simultaneously were considered as reliable in this study. Hence, according to these criteria, 53 unique SNPs, along with 46 SNPs within candidate genes that exhibited significant associations with root morphological traits at the critical threshold of -log_10_(*P*) ≥ 6.0 were obtained (Table 6; Fig. 4). These abovementioned SNPs were distributed all over the 10 maize chromosomes and individually explained between 5.10 and 30.2 % of phenotypic variation (Table 6). When analyzing significant SNPs that were detected throughout different stages, 17 SNPs were repeatedly detected from at least two stages along with 3 stable SNPs scanned across all the three growth stages (Table 6; Fig. 4). Our study regarded these SNPs as of great interest for further breeding purposes. Comparing the results across the different GWAS approaches, 34, 19, and 1 SNPs were identified by EMMAX, FarmCPU, and MLM, respectively (Table 6; Fig. 4). The SNP with the lowest p-value was located on chromosome 7, position 58,218,452 (-log_10_(*P*) = 14.95, R^2^ = 30.2 %), and was associated with RBN and SUA. This SNP was detected by FarmCPU stably across V1 and V3 stages. The SNP on chromosome 2 (S2_1707072, -log_10_(*P*) = 8.36, R^2^ = 25.1 %) was simultaneously detected by two different methods (EMMAX, MLM) at V2 stage. In regards of significant SNPs controlling multiple traits, two SNPs located on chromosomes 1 and 5 (S1_227871089, S5_82882718) were substantially linked to three root traits including ROV (-log_10_(*P*) = 6.06, 14.10, R^2^ = 14 %, 22.8 %), RDW(-log_10_(*P* ) = 6.10, 6.87, R^2^ = 14 %, 6.3 %), and SUA (-log_10_(*P*) = 7.02, 14.10 R^2^ = 12.1 %, 22.8 %), respectively. Another SNP on chromosome 2 (S2_43293834, -log_10_(*P*) = 6.89, R^2^ = 11 %) was also significantly linked with three different root traits, including RBN, ROV and SUA. The Q-Q (quantile-quantile) plots of all traits at all stages are shown in Additional file 5: Figure S1.

**Table 6:** Potential significant SNPs associated with root related traits

Traits	Position	Method^a^	Stage	Chr		P-value	-log_10_(P)	R^2^	Genotype
ARD	209,661,144	2	V2		Chr3	8.51E-08	7.07	0.100	G/A
ARD	159,805,368	2	V2		Chr6	8.32E-07	6.08	0.111	T/G
RBN	72,599,741	1	V1,V3		Chr1	3.31E-07	6.48	0.101	T/G
RBN	127,352,056	1	V2		Chr1	2.95E-07	6.53	0.115	T/C
RBN	1,707,072	1,3	V2		Chr2	4.37E-09	8.36	0.251	G/A
RBN	192,996,724	1	V2		Chr2	7.59E-08	7.12	0.151	C/A
RBN	195,707,091	1	V2		Chr2	6.17E-08	7.21	0.152	C/T
RBN	2,564,296	1	V2		Chr2	1.86E-07	6.73	0.108	C/T
RBN	32,824,965	1	V2		Chr2	6.76E-08	7.17	0.107	G/C
RBN	171,057,172	2	V2		Chr3	4.68E-07	6.33	0.101	G/A
RBN	122,501,344	1	V1,V3		Chr4	8.91E-07	6.05	0.121	C/T
RBN	87,176,006	1	V1,V3		Chr5	7.08E-07	6.15	0.123	C/T
RBN	221,805,144	2	V2		Chr5	9.77E-07	6.01	0.134	G/A
RBN	178,455,944	2	V2		Chr5	2.29E-07	6.64	0.140	C/T
RBN	205,892,847	2	V2		Chr5	2.82E-07	6.55	0.122	T/C
RBN	128,905,260	1	V2		Chr6	8.32E-07	6.08	0.114	G/A
RBN	162,388,475	2	V2		Chr6	7.08E-07	6.15	0.121	G/A
RBN	88,347,963	1	V1,V3		Chr8	7.41E-07	6.13	0.138	T/C
RBN	145,938,150	2	V2		Chr9	5.25E-07	6.28	0.150	G/A
RBN, SUA	58,218,452	2	V1,V3		Chr7	1.12E-15	14.95	0.302	A/G
RBN,ROV, SUA	43,293,834	1	V1,V2		Chr2	1.29E-07	6.89	0.110	G/A
RDW	227,871,089	1	V2		Chr1	7.94E-07	6.10	0.140	C/T
RDW	82,567,249	1	V2		Chr1	7.76E-07	6.11	0.140	C/G
RDW	2,246,081	2	V2		Chr10	1.29E-11	10.89	0.223	G/A
RDW	191,539,297	2	V1,V3		Chr5	7.94E-07	6.10	0.160	G/C
RDW	82,882,718	2	V1,V3		Chr5	1.35E-07	6.87	0.063	C/A
RDW	118,512,703	2	V2		Chr7	9.55E-07	6.02	0.113	A/G
RDW,SUA	230,477,446	1	V1,V3		Chr1	8.91E-07	6.05	0.051	G/A
RDW/SDW	19,943,384	1	V2		Chr1	7.41E-09	8.13	0.160	C/T
ROV	227,871,089	1	V1,V2,V3		Chr1	8.71E-07	6.06	0.140	C/T
ROV	173,181,844	1	V2		Chr1	6.46E-07	6.19	0.120	G/A
ROV	150,754,726	1	V2		Chr2	8.32E-07	6.08	0.150	A/T
ROV	166,210,299	1	V2		Chr2	5.01E-07	6.30	0.122	C/T
ROV	21,486,113	1	V2		Chr2	1.70E-07	6.77	0.110	C/T
ROV	187,822,582	2	V2		Chr3	2.82E-12	11.55	0.140	C/T
ROV	12,060,838	2	V2		Chr3	1.00E-06	6.00	0.100	A/G
ROV	241,936,576	1	V1,V2,V3		Chr4	9.77E-07	6.01	0.052	C/G
ROV	118,806,068	1	V2		Chr5	9.55E-07	6.02	0.153	T/C
ROV	28,955,506	1	V2		Chr6	8.32E-07	6.08	0.054	G/A
ROV,SUA	82,882,718	2	V1,V2,V3		Chr5	7.94E-15	14.10	0.228	C/A
SUA	227,871,089	1	V2		Chr1	9.55E-08	7.02	0.121	C/T
SUA	1,707,072	1	V2		Chr2	3.55E-07	6.45	0.200	G/A
SUA	2,610,094	1	V2		Chr2	2.57E-08	7.59	0.121	C/G
SUA	94,781,431	1	V2,V3		Chr3	7.94E-07	6.10	0.153	C/T
SUA	7,141,374	2	V1,V3		Chr5	6.46E-08	7.19	0.152	G/A
SUA	217,144,020	2	V1,V3		Chr5	1.55E-12	11.81	0.160	A/T
SUA	119,718,590	1	V2		Chr5	5.50E-07	6.26	0.150	C/T
SUA	179,029,112	1	V2		Chr7	2.40E-07	6.62	0.145	C/T
TRL	72,599,769	1	V1,V3		Chr1	4.68E-08	7.33	0.100	T/C
TRL	111,734,317	2	V2		Chr2	7.08E-07	6.15	0.128	A/G
TRL	59,237,040	1	V2		Chr3	5.37E-08	7.27	0.134	G/A
TRL	241,936,576	1	V1,V3		Chr4	2.95E-07	6.53	0.061	C/G
TRL	128,905,254	1	V2		Chr6	5.13E-07	6.29	0.106	G/A

*MAF *minor allele frequency, *R*^2^ phenotypic contribution, *Chr *chromosome, *RDW *root dry weight, *RDW/SDW *root per shoot dry weight, *TRL *total root length, *SUA *surface area, *ARD *average root diameter, *ROV *root volume, *RBN *root branching number

^a^method 1-3 refers to EMMAX, FarmCPU, and MLM, respectively

### Candidate genes and functional annotations

A total of 46 genes, along with 41 genes with SNPs inside, were found showing associations with the seven root architectural traits (Table 7). The candidate gene Zm00001d019766 was found only 5.54 kb away from the most significant SNP detected in this study located on chromosome 7 (S7_58218452) and associated with RBN and SUA. This gene was predicted to encode a RING/U-box superfamily protein. The gene model Zm00001d032473 on chromosome 1 (S1_227871089 for ROV, RDW, and SUA located within exon of the candidate gene) was predicted to confer a nonsynonymous mutation associated with CDPK-related kinase 3. The candidate genes Zm00001d029482 (S1_72599741 and S1_72599769 within the candidate gene) and Zm00001d037546 (S6_128905260 and S6_128905254 within the candidate gene) located respectively on chromosomes 1 and 6 contained two significant markers found for two traits, TRL and RBN. The gene model Zm00001d029482 was predicted to encode a NAD (P)-binding Rossmann-fold superfamily protein. Gene model Zm00001d005925 (SNP S2_192996724 for RBN located within the candidate gene) encodes a phosphoglucose isomerase protein with various pathways including GDP-mannose biosynthesis, gluconeogenesis I, glycolysis I (from glucose 6-phosphate), starch biosynthesis, and sucrose biosynthesis I. Gene model Zm00001d017279 on chromosome 5 (SNP S5_191539297 for RDW located within the candidate gene) encodes a phenylalanine ammonia-lyase protein associated with trans-cinnamoyl-CoA biosynthesis pathways. Gene Zm00001d038676 located on chromosome 6 (SNP S6_162388475 for RBN located within the candidate gene) encodes xyloglucan 6-xylosyltransferase and xyloglucan glycosyltransferase associated with xyloglucan and biosynthesis. The details of all candidate genes associated with potential SNPs and the functional annotations are presented in Table 7.

**Table 7:** Candidate genes associated with potential SNPs and functional annotations

Gene_ID	Traits	Chr	SNP Position	Distance(bp)	Gene type	Functional annotation/biological pathway
Zm00001d043773	ARD	3	209,661,144	0	exon,synonymous	Putative clathrin assembly protein
Zm00001d038558	ARD	6	159,805,368	0	UTR3	cystatin3, CC3, Cysteine proteinase inhibitor 2,
Zm00001d029482	RBN	1	72,599,741	0	intronic	NAD(P)-binding Rossmann-fold superfamily protein
Zm00001d030376	RBN	1	127,352,056	0	intronic	ATP-dependent DNA helicase
Zm00001d001900	RBN	2	2,564,296	0	intronic	Probable cysteine protease RD21B
Zm00001d003119	RBN	2	32,824,965	0	intronic	Trafficking protein particle complex II-specific subunit 120 homolog
Zm00001d005925	RBN	2	192,996,724	0	UTR5	Glucose-6-phosphate isomerase 1 chloroplastic,
Zm00001d006030	RBN	2	195,707,091	0	intergenic	ENT domain-containing protein
Zm00001d042535	RBN	3	171,057,172	0	intronic	selenoprotein family protein
Zm00001d050783	RBN	4	122,501,344	0	exon,nonsynonymous	Molybdopterin synthase sulfur carrier subunit
Zm00001d015379	RBN	5	87,176,006	0	exon,nonsynonymous	Splicing factor arginine/serine-rich 12
Zm00001d016858	RBN	5	178,455,944	0	UTR3	Ankyrin repeat protein SKIP35
Zm00001d017751	RBN	5	205,892,847	0	exon,nonsynonymous	Pentatricopeptide repeat-containing protein (chloroplastic)
Zm00001d018496	RBN	5	221,805,144	0	exon,synonymous	Pumilio homolog 4
Zm00001d037546	RBN	6	128,905,260	0	intronic	unknown
Zm00001d038676	RBN	6	162,388,475	0	exon,synonymous	Probable xyloglucan glycosyltransferase 12,
Zm00001d009896	RBN	8	88,347,963	0	exon,nonsynonymous	unknown
Zm00001d047946	RBN	9	145,938,150	0	intronic	Cell cycle checkpoint protein RAD17
Zm00001d003405	RBN, ROV, SUA	2	43,293,834	5823	intergenic	Bifunctional inhibitor/lipid-transfer protein/seed storage 2 S albumin superfamily protein
Zm00001d001841	RBN, SUA	2	1,707,072	0	intronic	unknown
Zm00001d019766	RBN, SUA	7	58,218,452	-5544	intergenic	RING/U-box superfamily protein
Zm00001d029683	RDW	1	82,567,249	0	exon,nonsynonymous	irregular pollen exine1
Zm00001d017279	RDW	5	191,539,297	0	exon,nonsynonymous	Phenylalanine ammonia lyase7
Zm00001d020485	RDW	7	118,512,703	0	UTR5	Golgi SNAP receptor complex member 1
Zm00001d023292	RDW	10	2,246,081	0	exon,synonymous	Trigger factor-like protein TIG Chloroplastic
Zm00001d015290	RDW, ROV, SUA	5	82,882,718	2998	intergenic	Adagio protein 1
Zm00001d032558	RDW, SUA	1	230,477,446	20,619	intergenic	unknown
Zm00001d028001	RDW/SDW	1	19,943,384	0	UTR3	Unknown
Zm00001d031009	ROV	1	173,181,844	0	intronic	Protein tesmin/TSO1-like CXC 2
Zm00001d002751	ROV	2	21,486,113	0	intronic	Probable isoaspartyl peptidase/L-asparaginase 3
Zm00001d004960	ROV	2	150,754,726	0	intronic	2-isopropylmalate synthase 1 chloroplastic
Zm00001d005264	ROV	2	166,210,299	0	exon,synonymous	Tetratricopeptide repeat (TPR)-like superfamily protein
Zm00001d039693	ROV	3	12,060,838	0	intronic	Protein RST1
Zm00001d043059	ROV	3	187,822,582	0	UTR5	Protein FATTY ACID EXPORT 3 chloroplastic,
Zm00001d015779	ROV	5	118,806,068	0	intronic	14-3-3-like protein GF14 omega
Zm00001d035487	ROV	6	28,955,506	0	intronic	E3 SUMO-protein ligase SIZ1
Zm00001d032473	ROV, RDW, SUA	1	227,871,089	0	exon,nonsynonymous	CDPK-related kinase 3
Zm00001d053827	ROV, TRL	4	241,936,576	-2034	intergenic	BEACH domain-containing protein B
Zm00001d001901	SUA	2	2,610,094	0	UTR3	Reticulon-like protein B11
Zm00001d041070	SUA	3	94,781,431	0	intronic	5-methylthioadenosine/S-adenosylhomocysteine deaminase,
Zm00001d013252	SUA	5	7,141,374	0	UTR3	60 S ribosomal protein L13a-1
Zm00001d015788	SUA	5	119,718,590	0	UTR3	proteasome component4, 26 S protease regulatory subunit S10B
Zm00001d018235	SUA	5	217,144,020	0	intronic	unknown
Zm00001d022502	SUA	7	179,029,112	0	intronic	Exocyst complex component Sec. 8
Zm00001d029482	TRL	1	72,599,769	0	intronic	NAD(P)-binding Rossmann-fold superfamily protein
Zm00001d004438	TRL	2	111,734,317	0	intronic	Pullulanase-type starch debranching enzyme1
Zm00001d040704	TRL	3	59,237,040	0	exon,nonsynonymous	ATP-dependent DNA helicase
Zm00001d037546	TRL	6	128,905,254	0	intronic	unknown

*RDW *root dry weight, *RDW/SDW *root per shoot dry weight, *TRL *total root length, *SUA *surface area, *ARD *average root diameter, *ROV *root volume, *RBN *root branching number, *Chr *chromosome

### Expression levels analysis

**Fig. 5:**
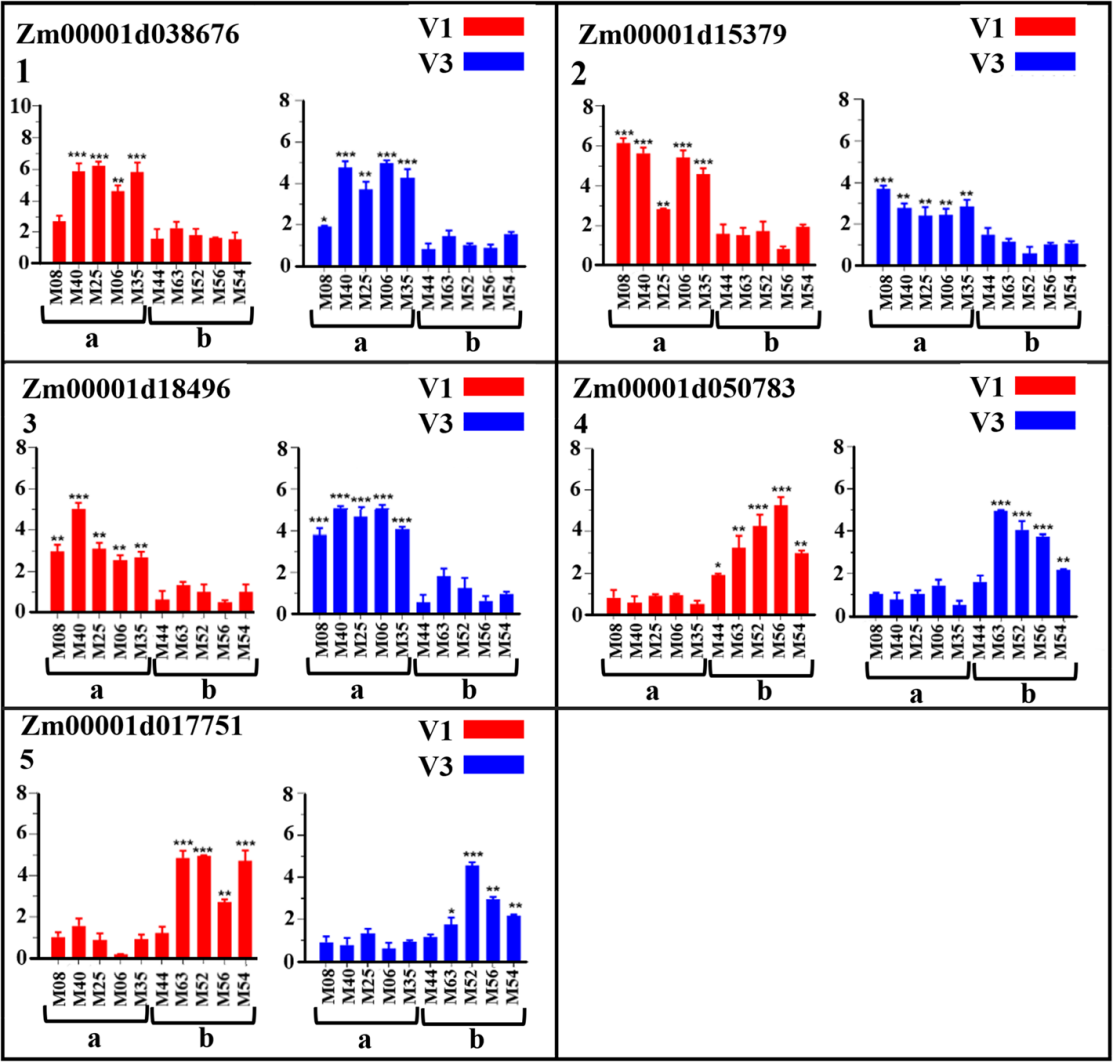
Relative expression levels (mean from three replicates) of five putative candidate genes (1 to 5) at V1 (in red bars) and V3 (in blue bars) growth stages in phenotypically extreme maize accessions for root branching number trait from the GWAS panel. Values of fold difference are shown in mean ± standard deviation (error bar). Relative expression levels were calculated using the 2^(-∆∆ct) method. 1, 2, and 3 are positive regulating genes while 4 and 5 are negative regulating genes. a and b stand for high and low root branching number accessions, respectively. ***, **, and * indicate the significance level for *P* < 0.001, *P* < 0.01 and *P* < 0.05, respectively

**Fig. 6:**
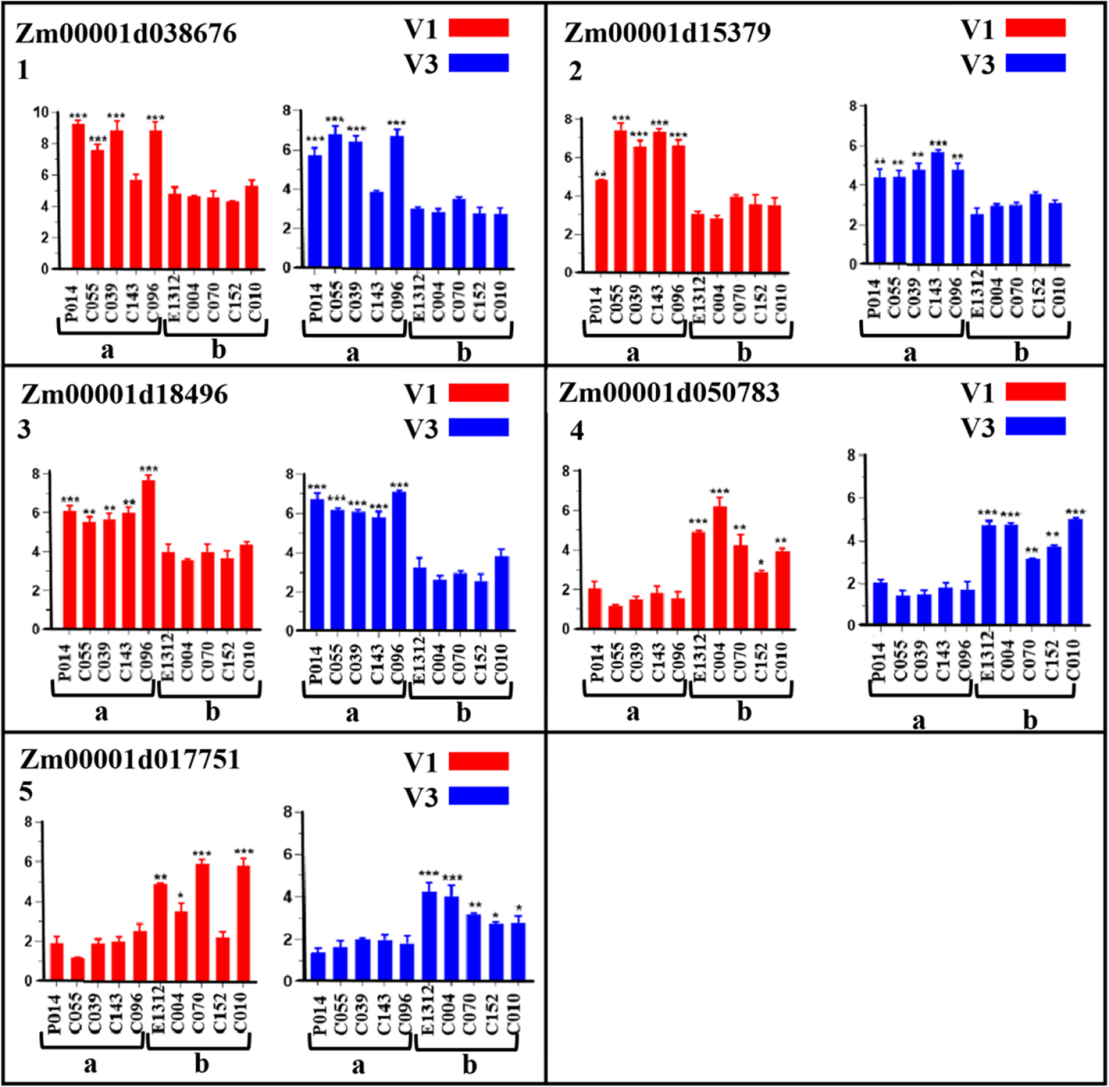
Relative expression levels (mean from three replicates) of five putative candidate genes (1 to 5) at V1 (in red bars) and V3 (in blue bars) growth stages in phenotypically extreme maize accessions for root branching number trait from the biparental population. Values of fold difference are shown in mean ± standard deviation (error bar). Relative expression levels were calculated using the 2^(-∆∆ct) method. 1, 2, and 3 are positive regulating genes while 4 and 5 are negative regulating genes. a and b stand for high and low root branching number accessions, respectively. ***, **, and * indicate the significance level for *P* < 0.001; *P* < 0.01 and *P* < 0.05, respectively

Five candidate genes including Zm00001d015379, Zm00001d050783, Zm00001d018496, Zm00001d038676, and Zm00001d017751 harboring significant SNPs (with phenotypic contribution rates greater than 12 %) within exonic regions were tested for expression levels using maize accessions with extreme root branching number differences from both GWAS and mapping panels at two growth stages (V1, V3). The relative expression level results obtained through qRT-PCR revealed that three candidate genes viz. Zm00001d038676, Zm00001d015379, and Zm00001d018496 acted as positive regulators for root branching number while two genes viz. Zm00001d050783 and Zm00001d017751 acted as negative regulators for root branching in both GWAS and mapping accessions (Figs. 5 and 6) at all the considered stages (V1, V3). The expression values expressed in fold change of genes Zm00001d038676, Zm00001d015379, and Zm00001d018496 in both GWAS and mapping accessions with high root branching number were significantly higher (*P* < 0.05, *P* < 0.01, *P* < 0.001) than those of accessions with low root branching number at all V1 and V3 stages (Figs. 5 and 6). In contrast, genes Zm00001d050783 and Zm00001d017751 displayed significantly lower expression levels in accessions with high root branching number as compared to low root branching number accessions (Figs. 5 and 6).

## Discussion

The use of natural variation approaches not only promotes the genetic basis of complex traits and the discovery of new regulators but also, can facilitate the identification of interesting alleles that can be further used to dissect the molecular mechanisms of the genes underlying the trait variation, which may be directly used for breeding purposes [4]. In this study, wide ranges of variations in terms of seedling root related traits were observed at both stages investigated. In regards to all traits, root length and branching number showed the largest phenotypic variations. Thus far, considerable natural phenotypic variations for root system architecture traits in various maize panels have been reported [2, 24, 31, 44, 46, 47]. Broad sense heritability estimates were relatively high with similar tendencies across stages. Recently, inline heritability ranges have been recorded in similar studies regarding maize seedling root traits at various growth stages/time-points both under field and controlled conditions [8, 15]. The traits like root volume, total root length, surface area, and root branching number were found tightly correlated in this current investigation. Positive significant associations between seedling and adult root traits were earlier reported by Abdel-Ghani et al. [17]. These findings were consistent with those observed by several investigators [2, 24, 44]. Root dry weight, surface area and total root length were stated to affect plant’s nutrients and water assimilation and absorption [48].

Most of the earlier root-related studies have been carried out using low-density genetic linkage maps involved commonly simple sequence repeat (SSR) or restriction fragment length polymorphism (RFLP), resulting in scanty inter-marker intervals not efficient enough for accurate QTL and gene identification [49]. In crops, association studies based on LD were previously stated as an effective approach for detecting and identifying SNPs or genes correlated with complex traits, such as roots [50]. GWAS use millions of single nucleotide polymorphisms (SNPs) markers to determine alleles associated with multiple traits in crops and identify genes controlling their expression [51]. In this analysis, a total of 1,490,007 consistent SNP markers were distributed among the ten maize chromosomes to directly mine SNPs and genes putatively associated with seven root architectural traits viz. RDW, RDW/SDW, TRL, SUA, ARD, ROV, and RBN across three vegetative stages (V1, V2, and V3) and using three GWAS models (MLM, EMMAX, and FarmCPU). Abundant polymorphisms and fast LD decay make maize an excellent crop for association studies [52]. The average LD decay distance was 5.2 kb, which specially enhances SNPs and genes mapping accuracy as compared to the reported densities for GBS-SNP markers ranging from 6.2 kb to 100 Mbp [8, 44] in recent maize root-trait QTL studies (last summary) [22]. These results are consistent with the reported average LD decay for inbred maize lines occurring within 1–10 kb [2, 53]. Based on significant SNPs (for multiple testing critical threshold of -log_10_(P) ≥ 6.0) identified within candidate genes or across at least two stages/methods simultaneously, 53 potential SNPs were detected. Comparatively, EMMAX and FarmCPU were the most efficient and accurate models for detecting 34 SNPs and 19 SNPs, respectively, while MLM model was the least efficient by detecting only one significant SNP. Due to its high stringency, MLM was earlier noted to create type II errors and cause false negatives [2, 44]. Recently, in a similar study, Sanchez et al. monitored fourteen SNPs through FarmCPU and four SNPs by MLM using a panel of 62,077 SNP markers [44]. It was previously indicated that various algorithmic approaches ought to be used to perform GWAS analysis in a real application for complex trait studies due to the limitation in a single model to detect polygenic variation associations [54–56]. A large number of root-related trait SNPs detected in this study are consistent with known genes recorded in previous findings. Using the results of fifteen root QTL studies from nine different bi-parental mapping populations, a meta-analysis study of QTLs recapitulated many putative MQTLs related to maize root development [57]. Interestingly, our detected SNPs were found to be in LD with four noteworthy loci involved in root development throughout developmental stages, namely S1_227871089 with Ax-2 (at bin 1.07), S2_32824965 and S2_43293834 with Rt-6 (at bin 2.04), S3_171057172, and S3_187822582 with Rt-7 (at bin 3.06), and S6_128905254 and S6_128905260 with Rt-13 (at bin 6.05) [57]. SNP S1_227871089 on chromosome 1 significantly associated with ROV, RDW and SUA housed within the candidate gene Zm00001d032473 predicted to encode a CDPK-related kinase 3 enzyme highly expressed in primary root growth [58–60]. This SNP was also in LD with the detected cluster QTLs qRDW_v3_-1-1 and qRDW/SDW_v3_-1-1 in addition to *qTRSA21-1*, *qTRL21-1* [19], and _*9d*_*RTN1-1* [24]. SNP S4-122501344 associated with RBN was located within gene Zm00001d050783 which codes molybdopterin synthase sulfur carrier subunit. This SNP was also in LD with the detected QTLS qSUA_v1_-4-1, qSUA_v2_-4-1, and qROV_v2_-4-1 in addition to the gene model GRMZM2G32186 [2] associated with several SNPs for root length, which showed high expression in the maize primary root at emergence and V1 stage [61]. Furthermore, SNP S4-122501344 was found to be within another reported gene (GRMZM2G153722) with high expression in seedling root and shoot [61] which contains nine other SNPs associated with root diameter and surface area [2]. SNPs S5_87176006 and S5_205892847 associated with RBN were located within gene models Zm00001d015379 and Zm00001d017751 which encode for an arginine/serine-rich protein 12 and a pentatricopeptide repeat-containing protein At2g15820 like, respectively. These SNPs were in LD with a QTL for crown root length in bin 5.04 detected by Liu et al. [15]. SNPs S6_128905254 and S6_128905260 significantly associated with TRL and RBN were found to be within the gene model Zm00001d037546 with currently unknown function. Its associated synonymous gene (GRMZM2G030235) highly expressed in maize seedling primary root at both VE and V1 stages [58, 61]. These SNPs were furthermore in LD with *qARL26-1*, *qARN26-1* [19] and a QTL for seminal root number in bin 6.05 reported by Liu et al. [15]. SNP S7_118512703 associated with RDW on chromosome 7 was found within Zm00001d020485, a gene encoding a Golgi SNAP receptor complex member 1. This SNP was also in LD with *qTRL17-1* [19] and _*9d*_*LRL7-1* [24]. Remarkably, the cluster qTRL_v1_-10-1 and qRBN_v1_-10-1 associated with total root length and root branching number at V1 stage collocated consistently with the QTL *qTRL*_*5d*_*-10-1* regulating total root length detected recently by Moussa et al. [45] within the same population using 5 days old seedlings. This chromosomal region can, therefore, be considered as a special noteworthy locus that can directly be used in marker-assisted selection programs.

When looking for SNPs within identified mutants or cloned genes for maize root development, rth6 [62] was putatively in LD with SNPs S1_127352056 and S1_173181844 associated with RBN and ROV; rth5 [63] was in LD with SNPs S3_171057172 and S3_187822582 associated with RBN and ROV; rum1 [64, 65] was in LD with SNP S3_209661144 associated with ARD; and rth2 [66] was in LD with SNPs S5_87176006, S5_118806068, S5_119718590, S5_205892847, and S5_82882718 associated with RBN, ROV, SUA, and RDW.

Gene expression profile can show whether the gene possesses a biological function [67]. However, several genes have been broadly determined through GWAS, but most of them are new genes without functional verification. Today, qRT-PCR is widely used to validate GWAS-detected gene expression with high accuracy and sensitivity [68, 69]. In this study, five potential genes were checked for expression levels using maize accessions with extreme root branching number differences. At all considered growth stages (V1, V3), three genes viz. Zm00001d038676, Zm00001d015379, and Zm00001d018496 acted as positive regulators for root branching number with significantly higher expression levels in high root branching number maize accessions. Conversely, two other genes viz. Zm00001d050783 and Zm00001d017751 with significantly lower expression in high root branching number accessions were found to act as negative regulating genes. The identified SNPs and evaluated genes by enriching our insights into the molecular mechanisms of maize root architecture, could thus be of great value for future marker-assisted breeding programs. Upcoming research will, however thoroughly elucidate the function of these genes in maize root growth and development.

## Conclusions

This study provides a comprehensive analysis of the genetic architecture of root traits in elite maize lines. A recombinant inbred line population and a genome-wide association study panel were used for mapping loci associated with maize root architectural traits at three developmental stages under standard conditions. QTL mapping using inclusive composite interval mapping identified eight QTLs for root traits on chromosomes 1(qRDW_v3_-1-1 and qRDW/SDW_v3_-1-1), 2 (qRBN_v1_-2-1), 4 (qSUA_v1_-4-1, qSUA_v2_-4-1, and qROV_v2_-4-1), and 10 (qTRL_v1_-10-1, qRBN_v1_-10-1), and each QTL explained 6.44–8.83 % of phenotypic variation. Genome-wide association study analysis identified 53 substantial SNPs through three GWAS models (EMMAX, FarmCPU, and MLM). The detected SNPs showed individual phenotypic contribution rates ranging from 5.10 to 30.2 % and were significantly associated with RBN (17), ROV (10), SUA (8), RDW (6), TRL (5), ARD (2), and RDW/SDW (1). Within the LD region of 5.2 kb for the significant SNPs, 46 candidate genes were identified. Five promising genes viz. Zm00001d038676, Zm00001d015379, Zm00001d018496, Zm00001d050783, and Zm00001d017751 were identified and successfully verified for expression level. The evaluated genes were shown to serve as positive/negative regulators for root branching in the spring maize lines. Thus, SNPs and underlying genes discovered in the present study could be critical for future marker-assisted breeding programs of high-efficient root systems in maize, as well as supporting the breeding of high-yielding maize varieties.

## Methods

### Plant materials, growth chamber experiment, and traits measurement

In this study, two genetic populations were used. The GWAS panel comprised 80 natural maize inbred lines covering more than 80 % planting area in Jilin Province (China). All the accessions are the same as defined in our earlier study [52]. The mapping population consisted of 179 RILs developed by a cross between the female parent P014 and the male parent E1312 and continuous inbreeding for 9 generations. P014 line was known to possess a larger and thicker root system with significantly higher root dry weight, total root length, surface area, projected area, average root diameter, and root tips as compared to E1312 line [45]. Genetically pure seeds from both populations were produced at the Jilin Agricultural University’s experimental field in summer 2019. A controlled growth chamber experiment was conducted using a completely randomized design (CRD) with three replications. The growth chamber parameters were: 28/25℃ temperature, 14/10 h (light/darkness) photoperiod, and 70/80 % relative humidity day/night, respectively. The light intensity was set at 200 µmol photons m^− 2^ s^− 1^. The seeds from both GWAS and mapping populations were directly planted in polyvinyl chloride (PVC) pipes (8 cm bottom diameter, 3.2 mm thickness, and 25 cm height) containing a mixture of sandy soil and vermiculite (2:1 ratio). Root architectural traits were investigated at three vegetative stages of maize growth namely V1 (one fully expanded leaf), V2 (two fully developed leaves), and V3 (three fully developed leaves) which approximately corresponded to 5, 15 and 25 days after emergence, respectively. For data collection, each PVC pipe consisted of one seedling was considered an experimental unit and the growth chamber independent trials were completed in February, April, and July 2020. All the experiments were repeated three times to increase the reliability of root quantitative traits measurements. The seedlings were cautiously removed from soil at each specific stage and washed with running water to eliminate the soil residues. For each seedling, the root system was separated from the shoot, and roots were scanned using a root scanner-based image (Perfection V800 Epson, resolution of 12,800 dots per inch (dpi: 5039.37 dots per cm)) then analyzed via DJ-GXG02 software [70]. If the collection of data could not be carried out within one single day, seedlings were kept in 30 % ethanol by swamping the roots and then placed in a cold chamber (4℃) with reference to Sanchez et al. [44]. A total of seven root related traits namely root dry weight, root to shoot dry weight, total root length, surface area, root volume, average root diameter, and root branching number were collected (Table 8). For dry weight biomass, root and shoot were recorded individually using an electronic weighing balance once drying in an oven set at 75℃ for at least 48 h to achieve the constant weight.

**Table 8:** Collected root related traits initials and illustrations

Trait Name	Abbreviations	Trait description
Root dry weight	RDW	Total root dry weight of the seedling in gram
Root to shoot dry weight	RDW/SDW	Root to shoot dry weight ratio in gram
Total root length	TRL	Cumulative length of the root system in cm
Surface area	SUA	Whole root system surface area in cm^2^
Root volume	ROV	Cumulative volume of all the roots in cm^3^
Average root diameter	ARD	Average diameter of the entire root system in mm
Root branching number	RBN	Total number of all the root tips

### Phenotypic analysis of root related traits

The descriptive analyses were carried out using Minitab17 program (Minitab Inc., State College, PA, USA). For every root related trait at each stage and in both populations, descriptive statistics including mean, standard deviation, maximum, minimum, skewness, kurtosis, and coefficient of variation were calculated. The broad-sense heritability (H^2^) was determined using variance components obtained via ANOVA as described by Pace et al. [2]. Normal distribution and Pearson coefficients of correlation among traits were also generated. Figures were produced using GraphPad Prism 8.0.1 (GraphPad Software, Inc., San Diego, CA).

### Genotyping, linkage map construction, and QTL mapping

The 179 RILs were genotyped by sequencing (GBS), and MSTmap was used for linkage analysis of marker data [71]. Linkage map was constructed based on automatic parameter settings with reference to Meng et al. [72]. Briefly, the markers were grouped at a LOD of ≥ 3.0, ordered, rippled, and then outputted to generate the linkage map. QTL IciMapping 4.1 software was used to perform QTL analysis using inclusive composite interval mapping for additive QTL (ICIM-ADD) [73]. The walking speed was 1.0 cM and the size of the windows was 5.0 cM. A LOD threshold peak score value of ≥ 2.5, which is commonly used in maize QTL mapping [49, 74], was set to declare significant QTLs. QTL additive effects and phenotypic variance explained (PVE) were also analysed.

### Next-generation sequencing of GWAS population genome

Genomic DNA was extracted from leaves for the 80 maize inbred lines using a cetyltrimethylammonium bromide (CTAB) protocol for plant tissues [75]. The different accessions were genotyped via next-generation sequencing at Novogene Biological Company (https://novogene.com/, Beijing, China). Briefly, for genomic libraries construction, the DNA samples were digested by sonication to a size of 350 bp, the genomics fragments were then subjected to end polishing and A-tailing afterward ligated to the full-length adapter for Illumina HiSeq PE150 platform (Illumina Inc., San Diego, CA, USA). Subsequently, the libraries were analyzed using Agilent 2100 Bioanalyzer and high-consistent sequencing data that show polymorphisms were then mapped to the B73 maize reference genome (RefGen_v3) using BWA software [76]. Duplicates were expurgated using SAMtoots [77]. Thus, 34,872,961 SNPs were obtained from next-generation sequencing analysis. Finally, using the Bayesian model mpileup of SAMtools with the criteria of SNP missing rate of < 10 % and minor allele frequency (MAF) of ≥ 5 %, a final total of 1,490,007 high-consistent SNP markers were obtained for genetic evolution and GWAS analyses.

### Population structure and linkage disequilibrium analysis

Based on a total of 1,490,007 high-consistent SNP markers, the principal component analysis (PCA) of individuals was performed using genome-wide complex trait analysis software tool (GCTA) [78]. The distances between the materials were inferred based on the distance matrix for phylogenetic tree contruction using TreeBeST program (http://treesoft.sourceforge.net/treebest.shtml/). Using PLINK [79], the decay of linkage disequilibrium measured in base pairs was calculated on each chromosome using an r^2^ value of 0.1 as a cut-off.

### GWAS analysis

In this study, to control false positive or spurious associations, three GWAS models were implemented, viz.: (1) Mixed Linear Model (MLM) [80], where PCA (Q) from population structure and kinship (K) were used as covariates; (2) Efficient Mixed-Model Association eXpedited (EMMAX), which outperforms both PCA and genomic control in correcting for sample structure with high statistical power [81] and; (3) Fixed and random model Circulating Probability Unification (FarmCPU), where PCA (as a fixed effect) and kinship (as a random effect) were used as covariates [82]. GWAS using the MLM model and EMMAX model was performed using the software TASSEL 5.0 [83], and FarmCPU method was performed using R FarmCPU package [82]. The threshold was defined based on the number of effective SNP markers. Multiple testing using simpleM program integrated in R, which calculates the number of informative SNP markers (Meff_G) was used to set the threshold. Briefly, a correlation matrix for all 1,490,007 SNPs was generated and the corresponding eigenvalues were calculated; a composite LD correlation was then calculated directly using GAPIT package and once this SNP matrix was obtained, Meff_G was calculated and this value was used to compute for the multiple testing threshold in the same way as the Bonferroni correction method, where the significance threshold (α = 0.05) was divided by the Meff_G (α /Meff_G) [2, 44]. Finally, after adjusting, *P* ≤ 0.000001(-log_10_ (*P*) ≥ 6) was set as the critical threshold to detect SNPs significantly associated with the different root morphological traits.

### Candidate gene identification

Candidate genes with SNP in coding regions and which could promote mutation were considered as high priority candidate genes. MaizeGDB (http://www.maizegdb.org/) and Gramene (https://www.gramene.org/) databases were used for predicting functional annotations of the candidate genes with reference to B73_RefGen_v4 [84, 85].

### Expression analysis

High priority candidate genes were chosen based on the existence of substantial SNPs within the exon of candidate genes and phenotypic contribution rates greater than 12 %. For expression analysis, ten maize accessions with extreme root branching number differences from both GWAS and biparental populations were chosen. There were M08, M40, M25, M06, M35, M44, M63, M52, M56, and M54 from the GWAS population. From the mapping population, there were P014 (female parent), E1312 (male parent), C055, C039, C0143, C096, C004, C070, C152, and C010. The roots were sampled at two different stages (V1 and V3). Total RNA was extracted using the Trizol method, cDNA was reverse transcribed the All-in-One First-Strand cDNA Synthesis kit (GeneCopoeia Inc., USA) following the standard protocol. Oligonucleotide primers were designed with PrimerPremier5.0 (http://www.premierbiosoft.com) (Additional file 6: Table S5). The qRT-PCR protocol was performed in a total volume of 20 µL: 2µL cDNA, 2 µL of each forward and reverse primer, 10 µL qPCR Master Mix, and 4µL ddH_2_O. The qRT-PCR Thermo cycling conditions were: initial denaturation at 95 °C for 30 s, 40 cycles of 95 °C for 5 s, annealing at 58 °C for 30 s, extension at 72 °C for 15 s, and an infinite hold at 10 °C. Leunig was used as the internal control gene and the relative expression levels were calculated using the 2^(-∆∆ct) method [86]. All experiments were conducted in triplicates at each specified growth stage.

## Supplementary Information

**Additional file 1: Table S1.** Phenotypic data of the seven root-related traits from the mapping population at V1, V2 and V3 stages.

**Additional file 2: Table S2.** Phenotypic data of the seven root-related traits from the GWAS panel at V1, V2 and V3 stages.

**Additional file 3: Table S3**. Physical positions of the identified QTLs.

**Additional file 4: Table S4.** List of all significant SNPs detected in this study and their associated genes.

**Additional file 5: Figure S1.** Q-Q (quantile-quantile) plots of all traits at V1, V2 and V3 stages.

**Additional file 6: Table S5.** List of oligonucleotide primers used for the qRT-PCR assay for the evaluated candidate genes.

## Data Availability

All data analyzed during this study are included in the supplementary information files, and genotypic data have been deposited in the Sequence Read Archive (https://www.ncbi.nlm.nih.gov/sra) under the accession number PRJNA495031.

## Abbreviations

GWAS: Genome wide association study
QTL: Quantitative trait loci
SNP: Single nucleotide polymorphism
RDW: Root dry weight
RDW/SDW: Root per shoot dry weight
TRL: Total root length
SUA: Surface area
ARD: Average root diameter
ROV: Root volume
RBN: Root branching number
SD: Standard deviation
CV: Coefficient of variation
H^2^: Broad-sense heritability
LD: Linkage disequilibrium
EMMAX: Efficient mixed-model association expedited
MLM: Mixed linear model
FarmCPU: Fixed and random model circulating probability unification
GBS: Genotyping by sequencing
NGS: Next-generation sequencing
QQ plots: Quantile-quantile plots
RILs: Recombinant inbred lines
ANOVA: Analysis of variance
PCA: Principal component analysis
