# Supplementary material for: Genome-wide association screening and verification of potential genes associated with root architectural traits in maize (Zea mays L.) at multiple seedling stages
Source: BMC Genomics. 2021 Jul 20;22:558. doi: 10.1186/s12864-021-07874-x (PMC8290564; doi:10.1186/s12864-021-07874-x)
Supplement: Supplementary file 3 — Additional file 3: Table S3. Physical positions of the identified QTLs. [file 12864_2021_7874_MOESM3_ESM.docx]

**Table S3.** Physical positions for the identified QTLs

| QTL | Chr | Marker interval | Physical interval (bp) | Bin |
| --- | --- | --- | --- | --- |
| qRDW_v3_-1-1 | 1 | Snp3292_Snp3298 | 90524812-90928885 | 1.05 |
| qRDW/SDW_v3_-1-1 | 1 | Snp3292_Snp3298 | 90524812-90928885 | 1.05 |
| qRBN_v1_-2-1 | 2 | Snp16808_Snp16675 | 237003419-239893573 | 2.10 |
| qSUA_v1_-4-1 | 4 | Snp25452_Snp25434 | 57105764-58177549 | 4.05 |
| qSUA_v2_-4-1 | 4 | Snp26234_Snp26219 | 88673462-89196026 | 4.05 |
| qROV_v2_-4-1 | 4 | Snp25161_Snp25085 | 41061698-44005387 | 4.05 |
| qTRL_v1_-10-1 | 10 | Snp62466_Snp62578 | 136073638-137457242 | 10.05-06 |
| qRBN_v1_-10-1 | 10 | Snp62466_Snp62578 | 136073638-137457242 | 10.05-06 |

RDW = root dry weight; RDW/SDW = root per shoot dry weight; TRL=total root length; SUA = surface area; ARD = average root diameter; ROV = root volume; RBN = root branching number; Chr = chromosome.
