# Supplementary material for: Genome-wide association screening and verification of potential genes associated with root architectural traits in maize (Zea mays L.) at multiple seedling stages
Source: BMC Genomics. 2021 Jul 20;22:558. doi: 10.1186/s12864-021-07874-x (PMC8290564; doi:10.1186/s12864-021-07874-x)
Supplement: Supplementary file 5 — Additional file 5: Figure S1. Q-Q (quantile-quantile) plots of all traits at V1, V2 and V3 stages. [file 12864_2021_7874_MOESM5_ESM.docx]

**
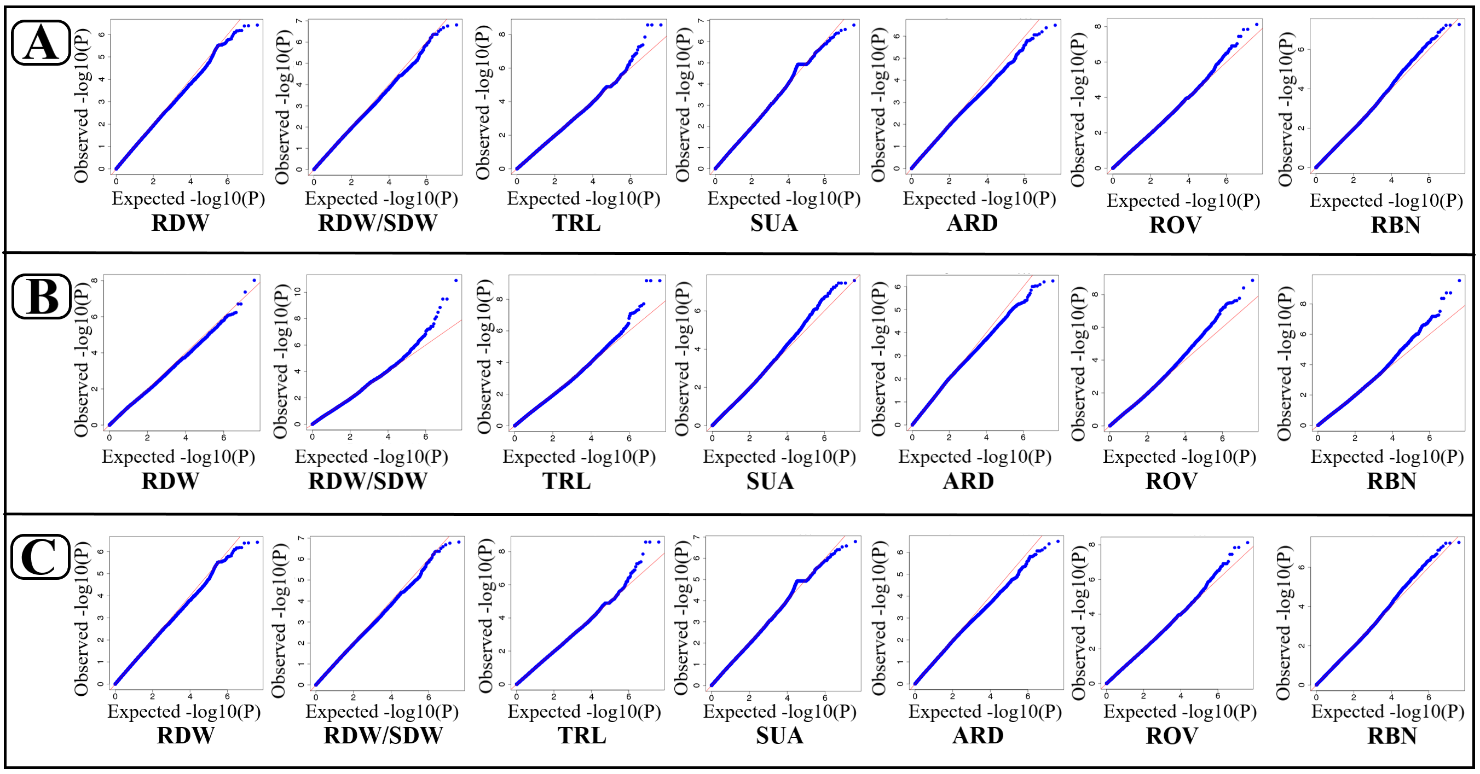
FIGURE S1-1.** Quantile-Quantile (Q-Q) plots of seven seedling root related traits in a panel of 80 elite inbred maize lines across three time points using EMMAX method.

A: results of V1 stage.

B: results of V2 stage.

C: results of V3 stage.


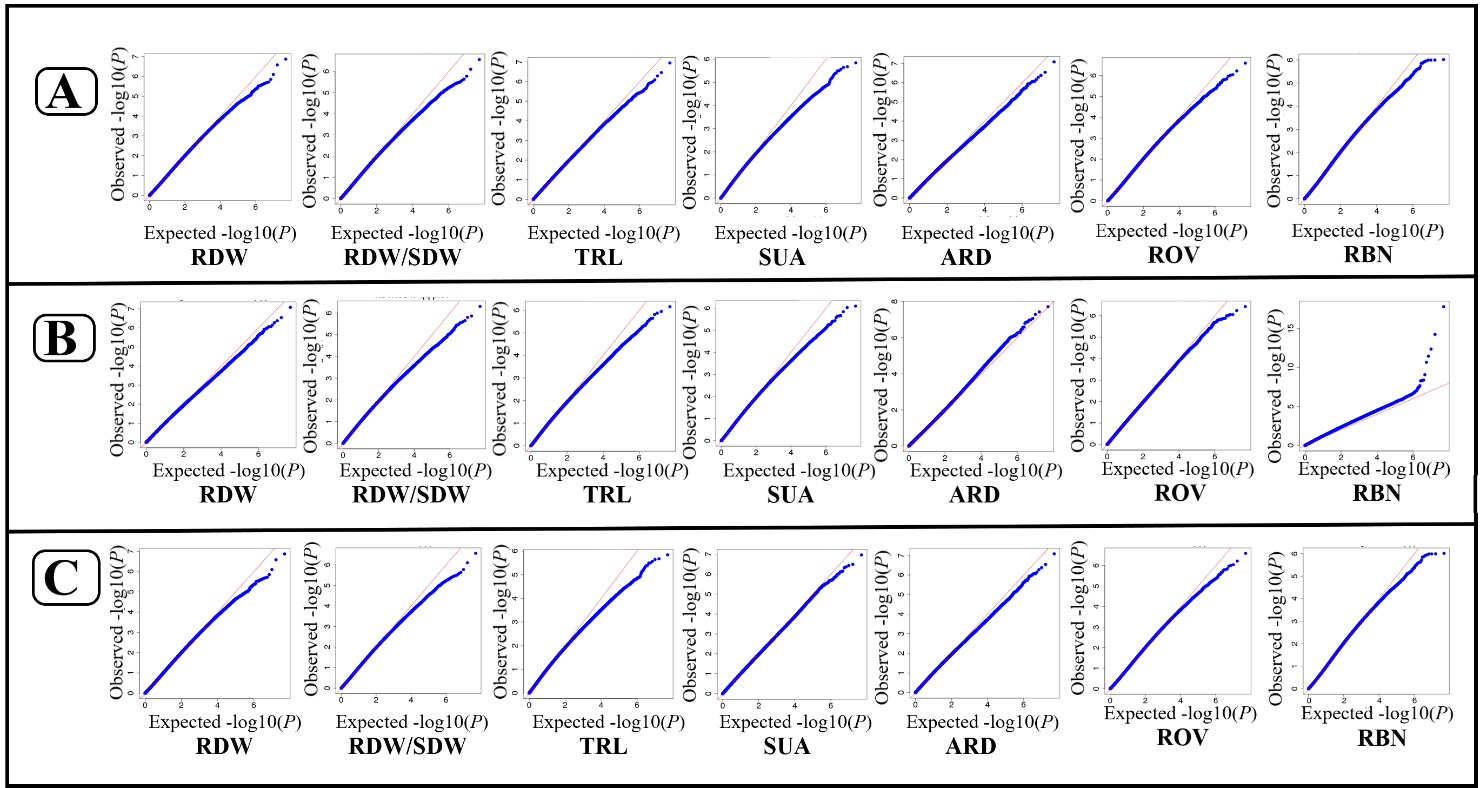


**FIGURE S1-2.** Quantile-Quantile (Q-Q) plots of seven seedling root related traits in a panel of 80 elite inbred maize lines across three time points using FarmCPU method.

A: results of V1 stage.

B: results of V2 stage.

C: results of V3 stage.

.


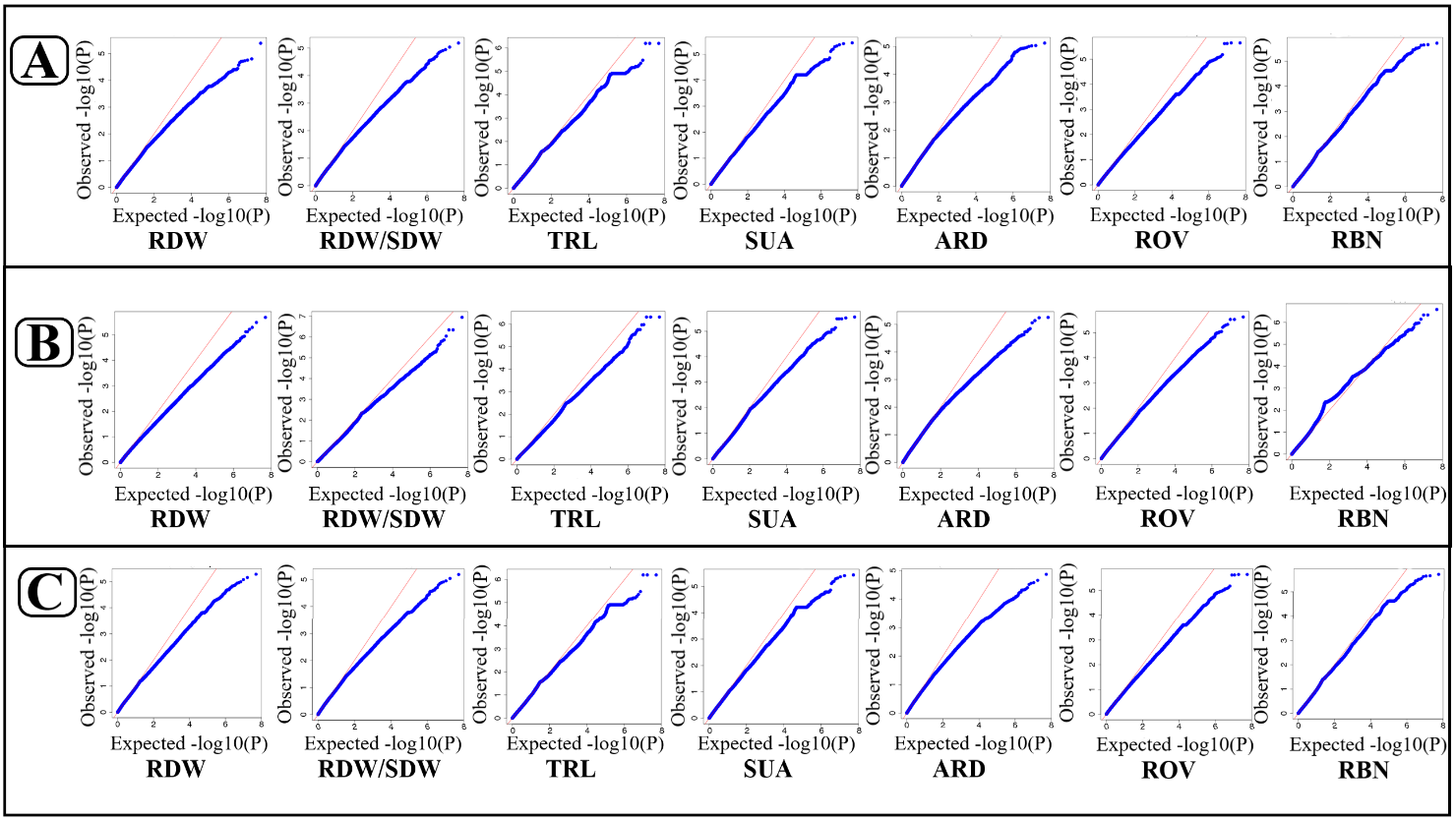


**FIGURE S1-3.** Quantile-Quantile (Q-Q) plots of seven seedling root related traits in a panel of 80 elite inbred maize lines across three time points using MLM method.

A: results of V1 stage.

B: results of V2 stage.

C: results of V3 stage.
