# Supplementary material for: Genome-wide association screening and verification of potential genes associated with root architectural traits in maize (Zea mays L.) at multiple seedling stages
Source: BMC Genomics. 2021 Jul 20;22:558. doi: 10.1186/s12864-021-07874-x (PMC8290564; doi:10.1186/s12864-021-07874-x)
Supplement: Supplementary file 6 — Additional file 6: Table S5. List of oligonucleotide primers used for the qRT-PCR assay for the evaluated candidate genes. [file 12864_2021_7874_MOESM6_ESM.docx]

**Table S5.** List of oligonucleotide primers used for the qRT-PCR assay for the evaluated candidate genes

| Gene_ID | Primer sequences (forward/reverse) | Product size |
| --- | --- | --- |
| Zm00001d038676 | AGATGCCCATGTGCAATGAGAGA | 103 |
|  | GAGATGCACCAGTTGGGGTTCTC |  |
| Zm00001d015379 | CAAGAAGGAGAGCAAGAGGAGGA | 159 |
|  | GCGTCCTTGTCTTTTCTCTTGCT |  |
| Zm00001d018496 | TAATGAGCACCTCAACCCAAGGC | 206 |
|  | TTTCACCCCTCCCCACTCTATGA |  |
| Zm00001d050783 | CAGTTCAGGACGACCTCCTTCAC | 106 |
|  | GTTCGCATCCAACACACAGATTC |  |
| Zm00001d017751 | CTGGAGGTTTTCGAGCTCGAAGA | 170 |
|  | AGGCAGTGGACGGTGATGTAGGT |  |
| GRMZM2G425377_T01 (Leunig) | TCCAGTGCTACAGGGAAGGT | 178 |
|  | GTTAGTTCTTGAGCCCACGC |  |
